# Preparation of Zeolitic Imidazolate Frameworks and Their Application as Flame Retardant and Smoke Suppression Agent for Rigid Polyurethane Foams

**DOI:** 10.3390/polym12020347

**Published:** 2020-02-05

**Authors:** Jiaji Cheng, Dan Ma, Shaoxiang Li, Wenjuan Qu, Dong Wang

**Affiliations:** 1 College of Environment and Safety Engineering, Qingdao University of Science and Technology, Qingdao 266042, China; madan7@126.com (D.M.);; 2 Shandong Engineering Research Center for Marine Environment Corrosion and Safety Protection, Qingdao University of Science and Technology, Qingdao 266042, China; 3 Shandong Engineering Technology Research Center for Advanced Coating, Qingdao University of Science and Technology, Qingdao 266042, China

**Keywords:** smoke suppression, flame retardancy, zeolitic imidazolate frameworks, rigid polyurethane foams

## Abstract

In order to reduce the fire risk of rigid polyurethane foams (RPUF), three kinds of zeolitic imidazolate frameworks (ZIFs) were prepared. The results of Fourier transform infrared spectroscopy (FTIR), scanning electron microscope (SEM and X-ray diffraction (XRD) showed that ZIFs were successfully prepared. The combustion test results showed that the heat and smoke production of the composite containing ZIFs was obviously reduced. In particular, the peak heat release rate (PHRR) of ZIF-8/RPUF decreased from 740.85 kW/m^2^ (Ref. RPUF) to 489.56 kW/m^2^, while the PHRR of ZIF-7/RPUF and ZIF-11/RPUF is 598.39 and 583.36 kW/m^2^, respectively. The addition of ZIFs improved the thermostability of the composite. The T50% of ZIF-8/RPUF, ZIF-7/RPUF and ZIF-11/RPUF increased to 364, 382 and 380 °C, respectively. The maximum light absorption of ZIF-7/RPUF and ZIF-11/RPUF was about 88%, which is higher than that of ZIF-8/RPUF (75%). The results of Raman spectroscopy showed that the I_D_/I_G_ value of Ref. RPUF is 2.96, while the I_D_/I_G_ value of ZIFs/RPUF reduces to less than 2.80. The main mechanism of ZIFs for reducing the fire risk of RPUF was the catalysis and incarbonization of ZIFs during combustion based on the results of thermogravimetric analysis and Raman spectroscopy of char residue.

## 1. Introduction

Metal–organic frameworks (MOFs), with a 3D network structure formed by the metal ions or clusters of metal ions connected to organic ligands [1,2], have attracted more and more attention due to their great potential in various applications, such as gas storage, separation, catalysis, carbon dioxide capture, drug delivery, sensing and photodynamic therapy [3,4,5].

Zeolitic imidazolate frameworks (ZIFs) are some of the most significant members of MOFs, typically composed of divalent metal cations and imidazolate bridging ligands. Twenty-five different ZIFs were synthesized by Banerjee and co-workers [6]. Members of a selection of these ZIFs with high porosity have excellent ability to capture and store CO_2_ from CO_2_/CO mixtures. Besides, hydrogen [7] and methane [8] can be selected from other gases by ZIFs.

The organic–inorganic hybrid nature of MOFs results in excellent compatibility with polymers. ZIFs also can be used for compatibilizing immiscible polymer blends [9]. The interfacial energy of immiscible polymers was reduced because of the selective location of ZIF-8 on two phase boundaries. Li et al. [10] reported uniform ZIFs/polyvinylidene fluoride membranes with attractive performance. The fabricated membranes with excellent hollow fiber structures and enhanced structural stability exhibited high H_2_ permselectivity. With the variety and diversity of functional polymers booming, it raises demands for more novel directions for integrating polymers and MOFs into various functional applications. 

Rigid polyurethane foams (RPUF) have been widely used in our lives because of their thermal conductivity, lightweight and construction convenience. However, the inherent flammability limits their applications for safety considerations [11,12,13]. The development of RPUF with excellent flame retardant property has been an active area of research. Flame retardants, for instance halogen, phosphorus, nitrogen, etc., are the most common additives for improving the thermal stability and fire resistance of RPUF [14,15,16]. However, these traditional flame retardants cannot meet the public demands for safety considerations. In recent years, MOFs were used as flame retardants in the polymer. Hou’s group [17] used a Co-based metal–organic framework of a phosphorus-containing structure (P-MOF) to enhance the fire safety of epoxy resin (EP). The combination of the adsorption and catalytic effect of P-MOF decreased the heat release rate (HRR) and smoke production, which provides a promising application of MOFs to enhance the fire safety of polymer materials. Different from most MOFs with weak thermostability, ZIFs own better thermal stability because the interaction between metal cations and the nitrogen atoms of imidazolate ligand is much closer than that of carboxybenzene. The effect of zeolitic imidazolate frameworks-8 (ZIF-8) on the fire risk of epoxy resin was investigated by Xu [18]. Compared with pure EP, the ZIF-8 reduced the heat release rate of composites and improved their limiting oxygen index (LOI) and UL94 vertical burning rating. Furthermore, the synergistic effect of ZIF-8 and distiller’s dried grains with solubles was shown by Xie et al. [19].

To verify the feasibility of the ZIFs’ application as flame retardants for RPUF, three kinds of ZIFs, zeolitic imidazolate frameworks-7 (ZIF-7), zeolitic imidazolate frameworks-8 (ZIF-8) and zeolitic imidazolate frameworks-11 (ZIF-11), were prepared and applied as flame retardants for RPUF in this paper. The thermal stability, smoke suppression and combustion behaviors were characterized by thermal gravimetric analysis, microscale combustion calorimeter and cone calorimetry. In addition, the feasible mechanisms of flame retardancy and smoke suppression for three different ZIFs were provided. Besides the flame retardant properties, the mechanical properties also play an important role in evaluating the performance of RPUF in applications. So, the mechanical test was conducted to assess the effects of ZIFs on the RPUF more comprehensive.

## 2. Experimental Section

### 2.1. Materials

Zn(NO_3_)_2_·6H_2_O was purchased from Damao Chemical Reagent Factory, Tianjin, China. C_4_H_6_O_4_Zn·2H_2_O was obtained from Shanghai Chemical Reagent Co., Ltd., Shanghai, China. Additionally, 2-methylimidazole (99% purity), benzimidazole and *N*,*N*-dimethylformamide were provided by Shanghai Macklin Biochemical Co., Ltd., Shanghai, China. Methylbenzene, ammonia water (concentration: 30 wt %), methyl alcohol and ethyl alcohol were purchased from Yantai Far Eastern Fine Chemical Co., Ltd., Yantai, China. Raw material A and B of RPUF were purchased from Safezike Co., Ltd., Xuzhou, China.

### 2.2. Preparation of ZIFs

#### 2.2.1. ZIF-7

Amounts of 0.595g of Zn(NO_3_)_2_·6H_2_O and 0.2365g benzimidazole were mixed into 45 mL of *N*,*N*-dimethylformamide, followed by magnetic stirring for 0.5 h. Then, the homogeneous dispersion was transformed into an autoclave (50 mL) and held at 130 °C for 24 h. The product was washed 3 times by centrifugal washing with methyl alcohol. Finally, the product was placed in a dryer for 12 h, and ZIF-7 was obtained.

#### 2.2.2. ZIF-8

An amount of 0.66 g Zn(NO_3_)_2_·6H_2_O was placed in 120 mL methyl alcohol, and 1.22 g 2-methylimidazole was placed in 110 mL ethyl alcohol; then, 2 kinds of solutions were mixed, stirred by a magnetic stirrer for 1 h under 40 °C. Subsequently, the production was washed 3 times by centrifugal washing with ethyl alcohol. Finally, the product was placed in a dryer for 12 h, and ZIF-8 was obtained.

#### 2.2.3. ZIF-11

An amount of 0.12 g benzimidazole, 4.6 g methylbenzene and 0.06 g ammonia water were mixed into 6.8 g ethyl alcohol, then 0.11 g C_4_H_6_O_4_Zn·2H_2_O was added. Then, the mixture was stirred by a magnetic stirrer for 3 h. Subsequently, the solution was washed 3 times by centrifugal washing and then washed once with ethyl alcohol.

### 2.3. Preparation of ZIFs/RPUF Composites

For the preparation of ZIFs/RPUF composites, 12 wt % ZIFs was placed in 35 wt % raw material A and stirred with an electric stirrer for 5 min. Then, 53 wt % raw material B was added with continuous stirring for 3 min. Then, the mixture was quickly poured into a mold. After 12 h, the specimen was obtained from the mold. Samples with ZIF-7, ZIF-8 and ZIF-11 were labeled as ZIF-7/RPUF, ZIF-8/RPUF and ZIF-11/RPUF.

### 2.4. Characterization

Fourier transform infrared spectroscopy (FTIR) test was conducted with the TENSOR27 type spectrometer (Bruker, Germany). The morphology of the samples was observed by using Hitachi-650 scanning electron microscope (SEM) (Hitachi, Japan). X-ray diffraction (XRD) measurement was tested with XRD-7000 (Shimadzu, Japan) at a scanning speed of 2°/min. Raman spectroscopy was tested by using a LabRam HR Evolution (Hobriba, France) and a wavelength of 514.5 nm.

Combustion properties of composites were performed on a microscale combustion calorimeter (MCC, GOVMARK) and a cone colorimeter (Suzhou, China) at 50 kW/m^2^. The thermal stability of the foams was performed by a DT-50 thermo-analyzer instrument from 30 to 800 °C. Smoke density was measured by a smoke density test (SDT) machine (TTech-GBT8627, Suzhou, China) according to GB/T 8627-2007. The compressive strength was measured with a universal electronic testing machine (CMT5305, Shenzhen, China) with a compressive rate of 1 mm/min. 

## 3. Results and Discussion

### 3.1. Characterization of ZIFs

**Figure 1:**
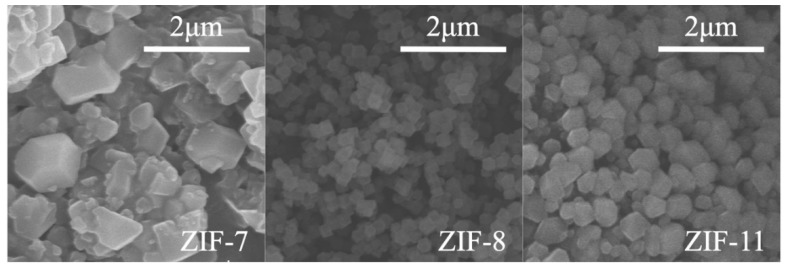
SEM images of zeolitic imidazolate framework (ZIF)-7, ZIF-8 and ZIF-11.

Figure 1 presents SEM images of ZIF-7, ZIF-8 and ZIF-11. It is obvious that ZIF-8 and ZIF-11 particles show the nanoscale size (0.15–0.35 μm and 0.40–0.55 μm, respectively) and better crystallization shape, while ZIF-7 exhibits a bigger size (1.0–1.3 μm).

**Figure 2:**
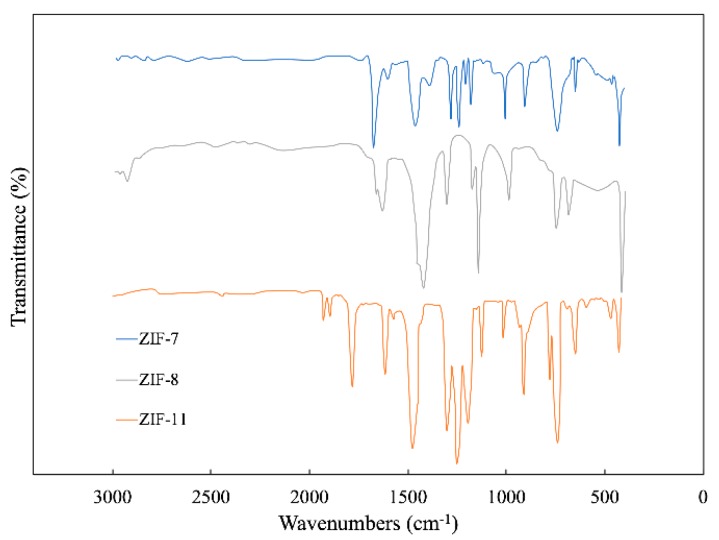
FTIR spectra of ZIF-7, ZIF-8 and ZIF-11.

Figure 2 shows the FTIR spectra of ZIF-7, ZIF-8 and ZIF-11. Excellent correspondence exists among the FTIR spectrum of ZIF-7, ZIF-8 and ZIF-11. For ZIF-8, the peaks at 2925 and 1630 cm^−1^ are stretching vibration absorption peaks of C-H and C=N in the imidazole ring, respectively. The absorption peaks at 1306, 1141 and 996 cm^−1^ are generated by the in-plane bending vibration of the imidazole ring. The peaks at 759 and 694 cm^−1^ are vibration absorption peaks of the out-of-plane bending vibration of the imidazole ring. The peak at 417 cm^−1^ is stretching vibration absorption peak of Zn–N [20] while the peaks in 2200–3000 cm^−1^ disappear, which can be observed in 2-methylimidazole. For ZIF-11, the peak that occurs at 421 cm^−1^ corresponds to Zn–N stretches [21]. The peaks at a wavenumber of 1471 and 1610 cm^−1^ are attributed to stretching of C–C in the benzimidazole aromatic ring [22], and the peaks between 600 and 1500 cm^−1^ are related to the entire benzimidazole ring stretching or bending [23]. 

The IR spectra of ZIF-7 revealed similar peaks in the region of 1500–400 cm^−1^. Two obvious peaks at a wavenumber of 1453 and 758 cm^−1^ are generated by C=C and C-H bonds, respectively, correspond to the benzene functional group of benzimidazole.

**Figure 3:**
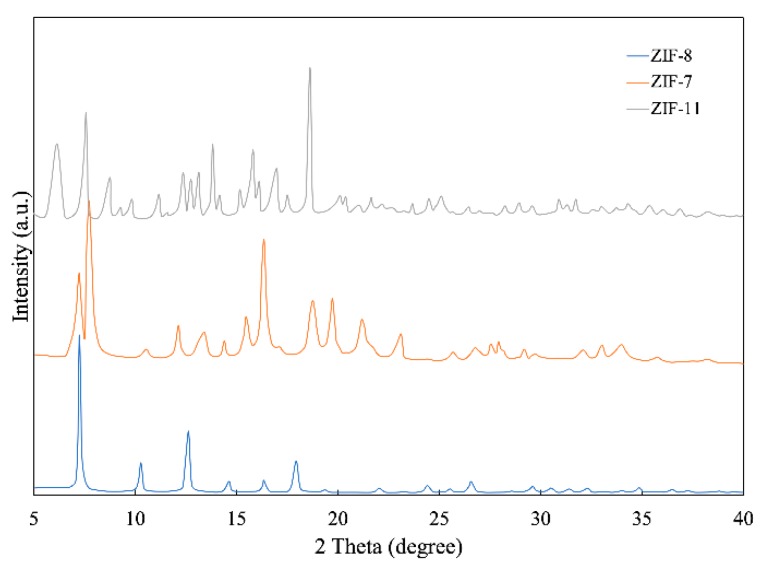
XRD patterns of ZIF-7, ZIF-8 and ZIF-11.

Figure 3 shows XRD patterns of ZIF-7, ZIF-8 and ZIF-11, in which all peaks are consistent with previous articles, confirming the successful synthesis of the metal–organic frameworks. For ZIF-8, the peaks at 2 theta = 7.52°, 10.28°, 12.64°, 14.64°, 16.35°, 17.9°, 22.03° and 24.41° are in compliance with previous articles [24]. Furthermore, for ZIF-7, the peaks are at 2 theta = 7.73 °, 12.13 °, 19.71°, 23.09° and 27.52°. For ZIF-11, the peaks are at 2 theta = 6.16°, 8.77°, 9.31°, 11.19°, 11.68°, 12.38°, 17.51° and 20.39°. The XRD patterns of ZIF-8 and ZIF-11 also agree with the simulated pattern [25] and those shown in the articles [26]. Compared with ZIF-7 and ZIF-11, ZIF-8 has better consistency with the simulated pattern, so the purity of ZIF-8 is much higher. 

### 3.2. Mechanical Properties of RPUF Composites

**Figure 4:**
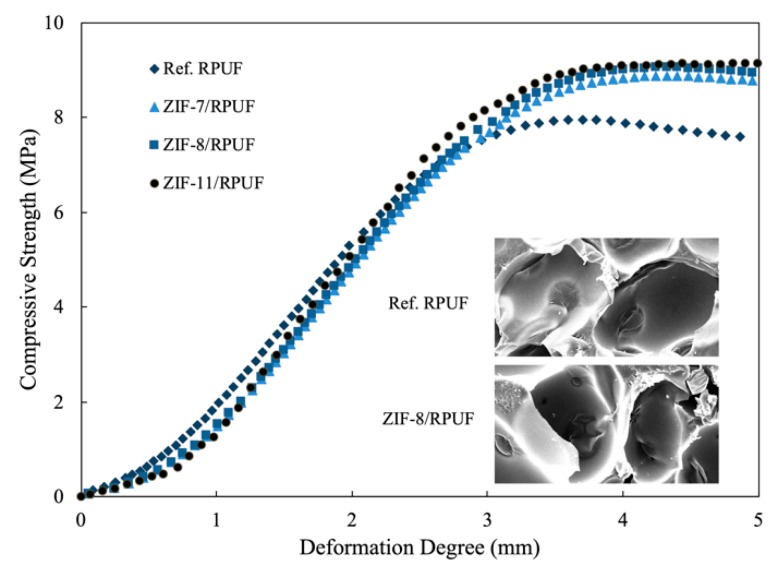
Compressive strength of Ref. polyurethane foams (RPUF), ZIF-7/RPUF, ZIF-8/RPUF and ZIF- 11/RPUF.

The compressive strength curves of composites and the SEM images are presented in Figure 4. The maximum compressive strength of composites is shown in Table 1. The pore size of Ref. RPUF is about 200 μm, and the morphology of ZIF-8/RPUF is similar to that of Ref. RPUF. It is hard to find the ZIFs in the SEM images of composites because the size of ZIFs is less than 1 μm. The compressive strength of composites rises slowly in the beginning period. With the increase of the deformation degree, the compressive strength increases rapidly and then enters a platform period because the cell walls begin to impair and the pore units start to fall down. The maximum compressive strength value of Ref. RPUF is 7.96 MPa. 

When ZIFs are added into the composite, the compressive strength value rises with the increase of the deformation degree. The compressive strength curves of ZIFs/RPUF are similar to that of Ref. RPUF when the deformation degree is less than 3.0 mm. This is because the compressive strength is too small to destroy the Ref. RPUF and ZIFs/RPUF. When the deformation degree is higher than 3.0 mm, Ref. RPUF is destroyed and the compressive strength value stops rising while the compressive strength values of ZIFs/RPUF increase to more than 8.9 MPa until the deformation degree reaches 4.0 mm. This is because the unique porous structures and high specific surface area of ZIFs result in how much energy is absorbed when the composite suffers from external stress [27,28]. Furthermore, compared with other flame retardant additives, for instance, ammonium polyphosphate, expansible graphite, et al., the interfacial strength between ZIFs and RPUF is stronger and ZIFs are easier to disperse evenly because ZIFs are built by organo-inorganic hybrid structure. So, ZIFs take an active influence on the mechanical properties of RPUF.

### 3.3. Thermal Behavior of ZIFs and ZIFs/RPUF

**Figure 5:**
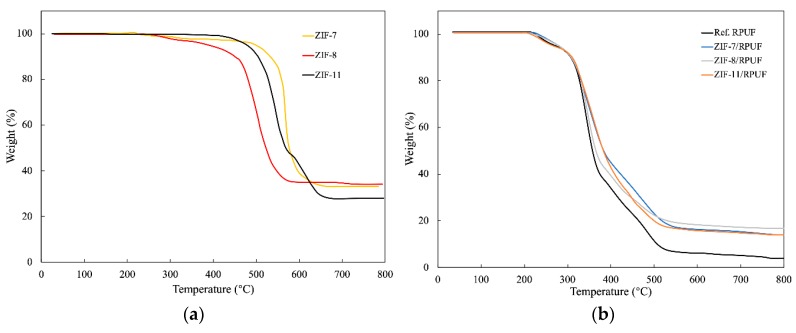
TGA curves of ZIFs (**a**) and ZIFs/RPUF (**b**).

The thermostability of ZIF-7, ZIF-8 and ZIF-11 is evaluated by TGA. As shown in Figure 5a, compared with ZIF-7 and ZIF-11, the mass loss of ZIF-8 appears at a lower temperature and shows a dramatic reduction at 500 °C. The drastic decrease in the mass loss of ZIF-7 and ZIF-11 appears between 550 and 600 °C. Figure 5b reveals the TG curves of ZIFs/RPUF. The weight of Ref. RPUF decreases dramatically from 300 °C and T_50%_ (the temperature when the mass of samples decreases to 50%) is 357 °C. When ZIFs are added, the T_50%_ of ZIF-8/RPUF, ZIF-7/RPUF and ZIF-11/RPUF increases to 364, 382 and 380 °C, respectively. This is because of the high degradation temperature of ZIFs. Furthermore, compared to Ref. RPUF, the char yields of ZIFs/RPUF at 800 °C are all increased because ZIFs promote char formation of the composite during the decomposition process. 

### 3.4. Combustion Properties of ZIFs/RPUF 

**Figure 6:**
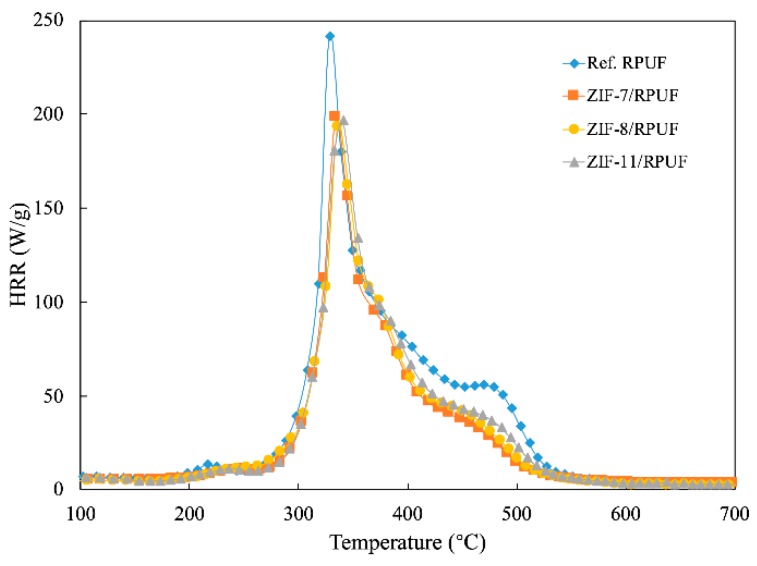
The heat release rate (HRR) curves of Ref. RPUF, ZIF-7/RPUF, ZIF-8/RPUF and ZIF-11/RPUF.

MCC is a convenient and effective laboratory test to evaluate the flame retardant property under controlled conditions [29]. The HRR of Ref. RPUF, ZIF-7/RPUF, ZIF-8/RPUF and ZIF-11/RPUF are presented in Figure 6. A decrease of about 17% in PHRR of ZIFs/RPUF is obtained compared to Ref. RPUF. When the temperature reaches 400 °C, the ZIFs begin to decompose and the gaps between Ref. RPUF and ZIFs/RPUF are enlarged. Besides HRR, the ignition temperature (IT) is another typical data. The IT of Ref. RPUF is 328 °C, while the IT of ZIFs/RPUF is around 340 °C, which is ascribed to the layered barrier effect. More char layers are promoted by the metal oxide generated by ZIFs, which hinders the heat transfer and the combustion of the composites. 

**Figure 7:**
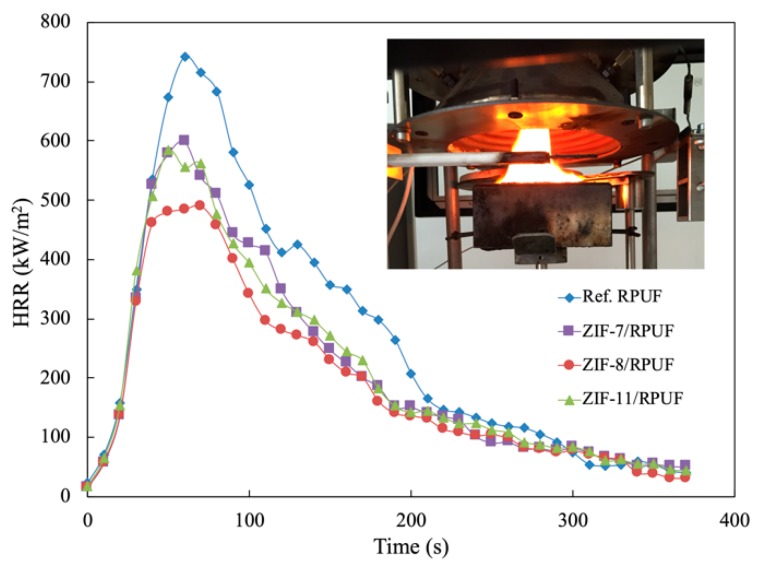
HRR curves of Ref. RPUF, ZIF-7/RPUF, ZIF-8/RPUF and ZIF-11/RPUF.

The cone calorimeter test is the most practical way to investigate the combustion behaviors of the polymer. The HRR, in particular, PHRR and total heat release (THR) are the significant data to evaluate potential fire hazards. As Figure 7 shows, the obvious decrease in the PHRR can be observed, which indicates the drop in fire hazards of composites. The PHRR is 740.85 kW/m^2^ for Ref. RPUF, while the PHRR of ZIF-7/RPUF and ZIF-11/RPUF is 598.39 and 583.36 kW/m^2^, respectively. Furthermore, the PHRR of ZIF-8/RPUF decreases by 33.9% to 489.56 kW/m^2^ compared with that of Ref. RPUF. This is mainly because ZnO produced in the combustion process of ZIFs could facilitate the generation of a char layer, thus inhibiting the release of the burning gas generated in the decomposition process of the composite. Agrawal added mineral fillers feldspar or kaolinite clay into RPUF in order to enhance the flame retardant properties [30]. The results showed that 10% of kaolinite clay or feldspar only decreases the PHRR by about 22%. 

**Figure 8:**
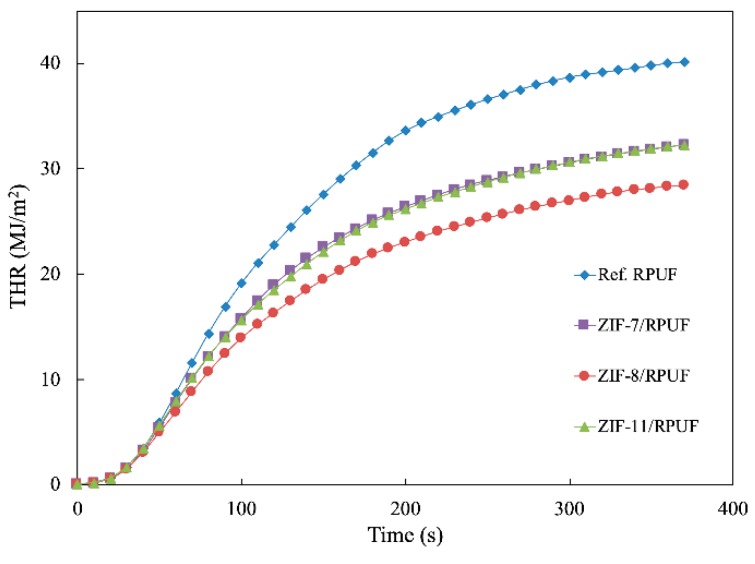
Total heat release (THR) curves of Ref. RPUF, ZIF-7/RPUF, ZIF-8/RPUF and ZIF-11/RPUF.

THR is an important parameter that reflects the condensed phase, which is shown in Figure 8. The THR of Ref. RPUF is 40.17 MJ/m^2^ at the end of the experiment, whereas ZIF-7/RPUF, ZIF-8/RPUF and ZIF-11/RPUF release a total heat of 32.22, 32.30 and 28.40 MJ/m^2^, respectively, indicating that a part of composites has not sufficient burned. It can be speculated that the decomposition of organic ligand in ZIFs produces the nonflammable gases, which decrease the flammable gas concentration and prohibit the combustion of composites [31]. Meanwhile, the catalytic effect of the metal oxide generated by ZIFs contributes to the thermal stability of char residue [32,33]. It is worth noting that the PHRR and THR of the ZIF-8/RPUF are 489.56 kW/m^2^ and 28.4 MJ/m^2^, respectively, which are much lower than those of ZIF-7/RPUF and ZIF-11/RPUF. This is because the metal oxide generated by ZIF-8 at about 500 °C according to Figure 5, which forms the physical barrier earlier than that of ZIF-7 and ZIF-11. 

**Figure 9:**
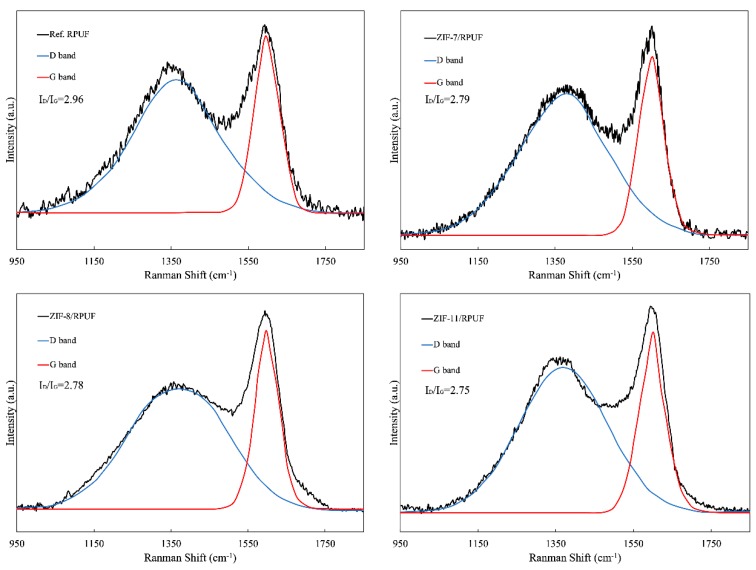
Raman spectra of char residue of Ref. RPUF, ZIF-7/RPUF, ZIF-8/RPUF and ZIF-11/RPUF.

Figure 9 gives the Raman spectra of the char residue of Ref. RPUF, ZIF-7/RPUF, ZIF-8/RPUF and ZIF-11/RPUF after burning in a cone calorimeter. It can be seen clearly from Figure 9 that the two obvious absorption peaks appear at about 1355 and 1585 cm^-1^ are named as the D and G peaks, respectively. The D peak is generated from symmetrical carbon atom vibration of amorphous carbon structure and the G peak is from crystalline graphitic carbon. As usual, the ratio of the integrated intensity of D and G peaks (I_D_/I_G_) is used to evaluate the graphitization degree of char [34,35,36]. Specifically, a lower the value of I_D_/I_G_ represents a higher degree of graphitization of a char residue, indicating that the char layer is denser and the thermal stability of char is higher. For Ref. RPUF, its I_D_/I_G_ value is 2.96, while the I_D_/I_G_ value of ZIFs/RPUF reduces to less than 2.80. This indicates that the addition of ZIFs is more conducive to the generation of char residue and the degree of graphitization, and isolates the transfer of heat and oxygen to provide the composite with better fire safety. Specifically, the metal oxide produced by the decomposition of ZIFs/RPUF has a catalyzing effect on the formation of the char layer and improves the formation of graphite carbon. So, oxygen is segregated and the volatilization of combustible gases is constrained, which contributes to the improvement of flame retardancy and smoke suppression of composite. Furthermore, Chai and et al. have proved that Al_2_O_3_ or MgO is decomposed at a specific temperature from aluminum trihydrate or magnesium hydroxide to cover the heat insulation, which benefits the formation of the carbonized layer [37].

**Figure 10:**
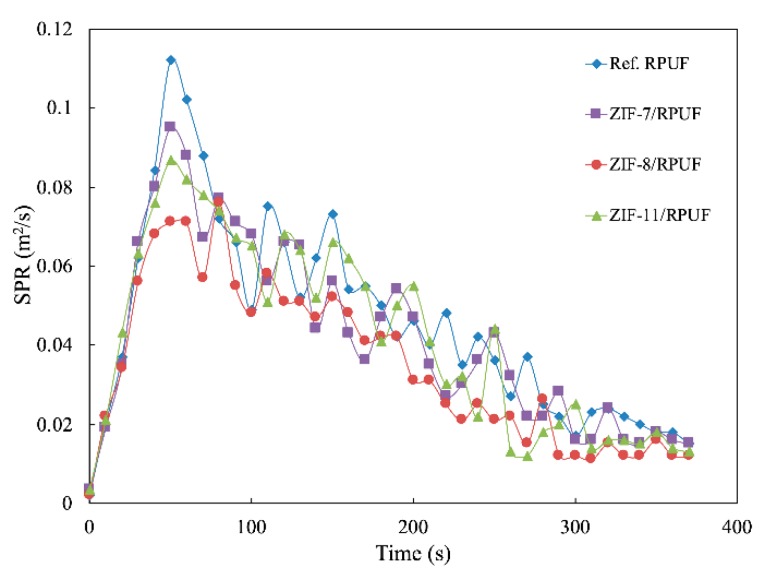
Smoke production rate (SPR) curves of Ref. RPUF, ZIF-7/RPUF, ZIF-8/RPUF and ZIF-11/RPUF.

**Figure 11:**
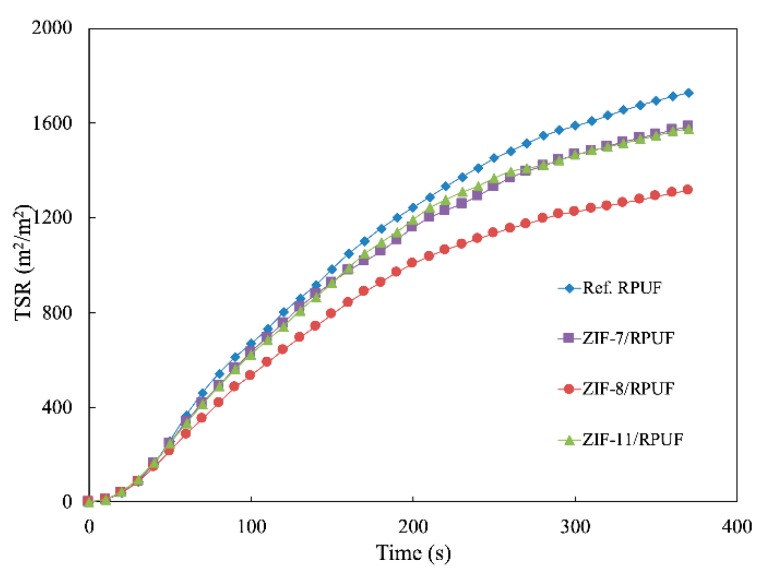
Total smoke release (TSR) curves of Ref. RPUF, ZIF-7/RPUF, ZIF-8/RPUF and ZIF-11/RPUF.

Besides HRR and THR, smoke production rate (SPR) and total smoke release (TSR) also are significant data to evaluate the hazards of the polymer during their combustion. It is known from Figure 10 and Figure 11 that the SPR of Ref. RPUF is large during burning and its peak of SPR and TSP are 0.112 m^2^/s and 1729.60 m^2^/m^2^, respectively. However, there is a significant reduction when ZIFs are added. The peak of SPR and TSP of ZIF-7/RPUF are reduced to 0.095 m^2^/s and 1585.20 m^2^/m^2^, respectively. ZIF-11 has a similar effect on the smoke production of composite, while ZIF-8 decreases the peak of SPR and TSP of the composite to 0.076 m^2^/s and 1316.10 m^2^/m^2^, respectively, corresponding to the 32.14% and 23.88% decrease compared with that of Ref. RPUF, indicating that ZIF-8 has a better smoke suppression effect than ZIF-7 and ZIF-11. This is because the metal oxide generated from the decomposition of ZIFs promotes the formation of the char layer, which prevents the release of smoke. The decomposition temperature of ZIF-8 is lower than that of ZIF-7 and ZIF-11. So, the metal oxide generated by ZIF-8 is earlier and benefits to the smoke suppression. Yuan et al. also proved that incorporation of 2 wt % NiO conspicuously increases the residual yield of RPUF nanocomposites by 63.8% due to its catalytic coupling effect [33].

**Figure 12:**
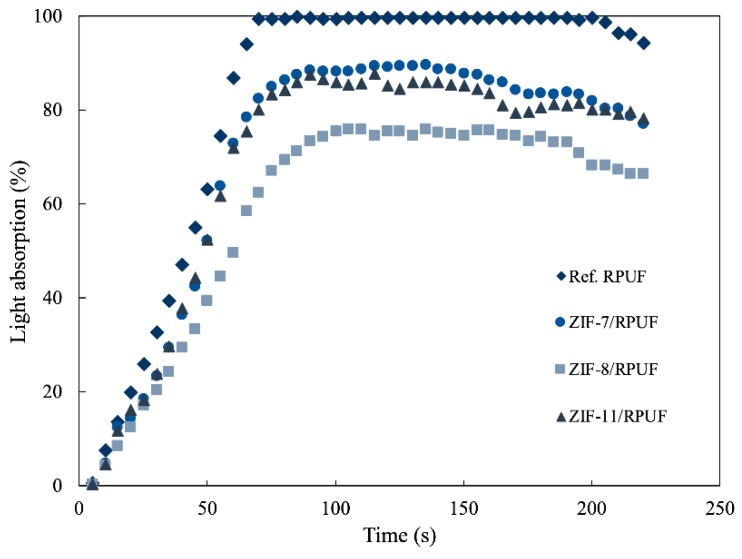
Light absorption of Ref. RPUF, ZIF-7/RPUF, ZIF-8/RPUF and ZIF-11/RPUF.

Figure 12 gives the light absorption of Ref. RPUF, ZIF-7/RPUF, ZIF-8/RPUF and ZIF-11/RPUF. A lot of smoke is produced by Ref. RPUF from the beginning of the experiment and light absorption rises to 100% at 70 s and keeps for 130 s. The addition of ZIFs decreases the rising speed of light absorption in the beginning. The maximum light absorption of ZIF-7/RPUF and ZIF-11/RPUF is about 88%, which is also higher than that of ZIF-8/RPUF (75%). Therefore, ZIFs suppress smoke efficiently and ZIF-8 has a better effect of restraining smoke than that of ZIF-7 and ZIF-11. 

## 4. Conclusions

In this study, three kinds of ZIFs (ZIF-7, ZIF-8 and ZIF-11) were synthesized and added into RPUF as flame retardants for the first time. SEM, FTIR and XRD showed that typical crystalline compounds were successfully prepared. The compressive strength test indicated that ZIFs take an active influence on the mechanical properties of RPUF. The maximum compressive strength value of Ref. RPUF is 7.96 MPa and the maximum compressive strength value of ZIFs/RPUF increases to more than 8.9 MPa. The degradation temperature of ZIF-7 and ZIF-11 is higher than that of ZIF-8 according to the result of TGA, while ZIFs improve the thermal stability of the composites and also promote char formation of the composite during the decomposition process. The T_50%_ of ZIF-8/RPUF, ZIF-7/RPUF and ZIF-11/RPUF increases to 364, 382 and 380 °C, respectively. According to the Raman spectra of char residue, metal oxide produced during the combustion of ZIFs plays a catalyzing role for the formation of char layer and improves the formation of graphite carbon, which prevents the matrix from further combustion. The I_D_/I_G_ value of Ref. RPUF is 2.96, while the I_D_/I_G_ value of ZIFs/RPUF reduces to less than 2.80. The results of combustion tests showed that a decreasing of about 17% in PHRR for ZIFs/RPUF is obtained compared to Ref. RPUF. The THR of Ref. RPUF is 40.17 MJ/m^2^ at the end of the experiment, whereas ZIF-7/RPUF, ZIF-8/RPUF and ZIF-11/RPUF release a total heat of 32.22, 32.30 and 28.40 MJ/m^2^, respectively, indicating that a part of composites has not sufficient burned. Besides, ZIFs suppress the smoke during the combustion of composites effectively. The maximum light absorption of ZIF-7/RPUF and ZIF-11/RPUF is about 88%, which is higher than that of ZIF-8/RPUF (75%).

The work depicts that the development of MOFs, such as ZIF-7, ZIF-8 and ZIF-11, is feasible and has great potential for reducing fire and smoke hazard of RPUF composites.

## Figures and Tables

**Table 1:** The maximum compressive strength of composites.

Test Item	Ref. RPUF [Standard Deviation]	ZIF-7/RPUF [Standard Deviation]	ZIF-8/RPUF [Standard Deviation]	ZIF-11/RPUF [Standard Deviation]
Maximum compressive strength	7.96 [0.085]	9.07 [0.076]	8.92 [0.140]	9.16 [0.078]
